# Comparison of the Efficacy and Safety of Single-Session OK-432 and Multiple-Session 99% Ethanol Sclerotherapy for Symptomatic Simple Hepatic Cysts

**DOI:** 10.3389/fmed.2022.737694

**Published:** 2022-07-15

**Authors:** Zhiqiang Mo, Fangfang Yang, Ling Lv, Jian He, Qin Gou, Xiaoming Chen, Wenhang Zhuang, Qicong Mai

**Affiliations:** ^1^Department of Interventional Radiology, Guangdong Provincial People's Hospital, Guangdong Academy of Medical Sciences, Guangzhou, China; ^2^Department of Medical Simulation Center, Guangdong Provincial People's Hospital, Guangdong Academy of Medical Sciences, Guangzhou, China; ^3^Department of Interventional Radiology, Shenzhen Traditional Chinese Medicine Hospital, Shenzhen, China

**Keywords:** hepatic disease, cysts, sclerotherapy, OK-432, clinical efficacy

## Abstract

**Purpose:**

This retrospective study aimed to compare the efficacy and safety of single-session OK-432 and multiple-session 99% ethanol sclerotherapy for symptomatic simple hepatic cysts.

**Methods:**

We reviewed patients who received aspiration sclerotherapy with OK-432 (group A) or 99% ethanol (group B) for symptomatic simple hepatic cysts at Guangdong Provincial People's Hospital from January 2013 to November 2019.

**Results:**

We included 42 patients in group A and 39 patients in group B. No significant difference was found in the mean volume of hepatic cysts between the two groups. The overall success rates were 92.9% (39 of 42 patients) in group A and 79.5% (31 of 39 patients) in group B (*P* = 0.08). The treatment success for cyst volumes <200 ml, 200–500 ml, and >500 ml was 100, 93.3, and 88.2% in group A, and 100, 84.6, and 57.1% in group B, respectively. The symptomatic relief rate in group A was higher than that in group B for cysts ≥500 ml (*P* = 0.049) and cysts <500 ml. For treatment-related complications, the incidence of pain at the injection site in group A was lower than that in group B.

**Conclusion:**

Single-session OK-432 sclerotherapy was safer and more effective than multiple-session 99% ethanol sclerotherapy for treating large cysts, although both treatments had similar effects on small cysts.

## Introduction

Simple hepatic cysts are fluid-filled cavities that arise from malformations of the ductal plate during embryonic development (1, 2). In most cases, simple hepatic cysts are asymptomatic. However, cysts tend to become voluminous over time owing to fluid production by secretory epithelial cells, which could lead to symptoms such as abdominal pain, distention, and impaired quality of life (1, 3, 4). For patients with symptomatic simple hepatic cysts, a treatment that aims to reduce cystic volume is indicated. In clinical practice, sclerotherapy is an optional treatment modality for symptomatic simple hepatic cysts, particularly for patients who are not eligible for surgery (3). In the past decade, studies on numerous sclerosing agents (e.g., ethanol, acetic acid, and tetracycline) for sclerotherapy have been published (5–8). Although these studies generally showed good clinical outcomes, sclerotherapy has been considered ineffective because of the high recurrence rate (9, 10). Several authors reported that multiple-session treatments that increase instilled volume and exposure time of sclerosing agents show better results in terms of recurrence (11–13). However, multiple sessions may result in troublesome repeat procedures, considerable risk of adverse events, and time-consuming hospitalization. Therefore, a single-session sclerosing agent that provides an excellent clinical response would optimize sclerotherapy.

OK-432 (Picibanil, Chugai Pharmaceutical Co., Tokyo, Japan), which is a lyophilized mixture of a low-virulence Su strain of *Streptococcus pyogenes*, has been used as a new sclerosing agent (14). Ogita et al. (15) reported that injection of OK-432 probably damages the endothelial lining, which causes obliteration of the cavity and prevents further accumulation of fluid in the lesion (15). Several authors have demonstrated that OK-432 has a high therapeutic effect and low recurrence rate without substantial adverse effects for treating cystic osteitis fibrosa, cystic hygroma, and malignant exudate in the thoracic cavity (16, 17). Based on these studies, we presumed that OK-432 might be beneficial for treating symptomatic simple hepatic cysts.

In this study, we aimed to compare the efficacy and safety of single-session OK-432 and multiple-session 99% ethanol sclerotherapy for symptomatic simple hepatic cysts. We analyzed the role of OK-432 and 99% ethanol on lesion regression and adverse reactions. This novel finding could be clinically significant for optimizing sclerotherapy for symptomatic simple hepatic cysts.

## Materials and Methods

### Study Population

In this retrospective cohort study, we reviewed patients who received aspiration sclerotherapy for symptomatic simple (non-parasitic, non-neoplastic) hepatic cysts at Guangdong Provincial People's Hospital from January 2013 to November 2019. A simple hepatic cyst was defined as an anechoic, unilocular fluid-filled space with imperceptible walls showing posterior enhancement on sonography and a well-demarcated water-attenuation lesion with no contrast enhancement on computed tomography (CT). The indication for aspiration sclerotherapy was based on the presence of symptoms that were likely to result from compression by a large hepatic cyst. Cyst volume was calculated by applying the following formula: V = π/6 × (length × width × depth).

Data on patients' clinical characteristics were collected. Informed consent was obtained from all participants. The Ethics Committee of Guangdong Provincial People's Hospital approved this study. All study protocols were approved by the institutional review board and carried out in compliance with the Declaration of Helsinki.

### Treatment Methods

On the day of their procedure, patients were positioned on the CT gantry to locate the lesion site, and 5 mm axial slices were obtained to delineate the lesion. After local infiltration anesthesia with 5–15 ml of 1% lidocaine, an 18 gauge puncture needle was used to puncture the cyst under CT guidance. After placing the puncture needle, a 0.96 mm J-tip guide wire was passed into the cyst. The puncture needle was then removed, and a 6-Fr pigtail catheter was inserted into the cyst over the guidewire. All cystic fluid was aspirated through a 6-Fr pigtail catheter. The first 10 ml of the aspirated fluid was sent for cytological and biochemical examinations.

For single-session OK-432 sclerotherapy (group A), OK-432 solution was prepared by dissolving 0.1 mg of OK-432 in 10 ml of 0.9% saline, and 0.1 mg of OK-432 per 10 ml of aspirated cystic fluid was injected into the cyst through a 6-Fr pigtail catheter. The 6-Fr pigtail catheter was then removed. The maximum injected volume of OK-432 was 100 ml.

For multiple-session 99% ethanol sclerotherapy (group B), 99% ethanol was injected into the cyst at a volume equal to 25% of the aspirated cystic fluid volume but never exceeded 50 ml in a single injection for safety reasons. The injected ethanol was completely re-aspirated after 20 min of sclerotherapy. Sessions were performed twice with an interval of 12 h. The catheter was left open for natural drainage between the sessions and then removed after the final session. The procedure was terminated if patients had severe abdominal pain at the injection site.

### Study Endpoints

**Figure 1:**
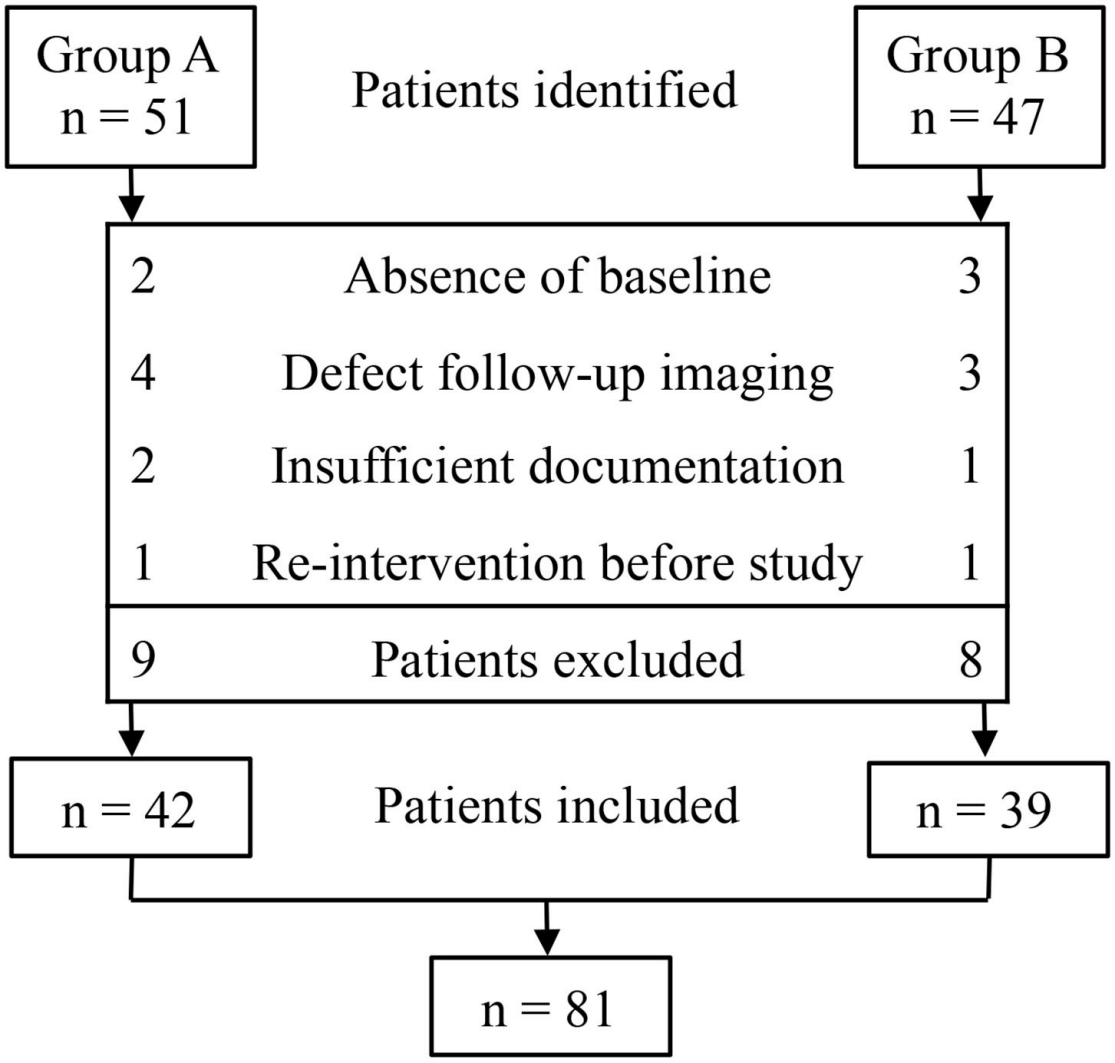
Flowchart: identification and selection of patients in groups A and B.

The primary endpoints of this study were proportional cyst volume reduction and symptoms. Patients were followed up every 3 months for 1 year by clinical assessment and CT imaging (Figure 1). To calculate the proportional cyst volume reduction, we used the maximum postoperative cyst volume and preoperative cyst volume. Complete regression of the hepatic cysts or >70% volume reduction with no symptoms was considered a successful treatment. A volume reduction of <70% and/or persistent symptoms were considered treatment failures. The secondary endpoint was treatment safety, including type, risk, and severity of complications following treatment within 1 year.

### Statistical Analysis

Continuous numerical variables were presented as mean ± standard deviation. Categorical variables were presented as frequencies (percentages). The chi-square test was used to investigate the association between categorical factors, and Student's *t*-test was used for continuous variables. Statistical analyses were performed using SPSS version 25.0 (International Business Machines, New York, USA). Statistical significance was set at *P* ≤ 0.05.

## Results

### Clinicopathological Characteristics

**Figure 2:**
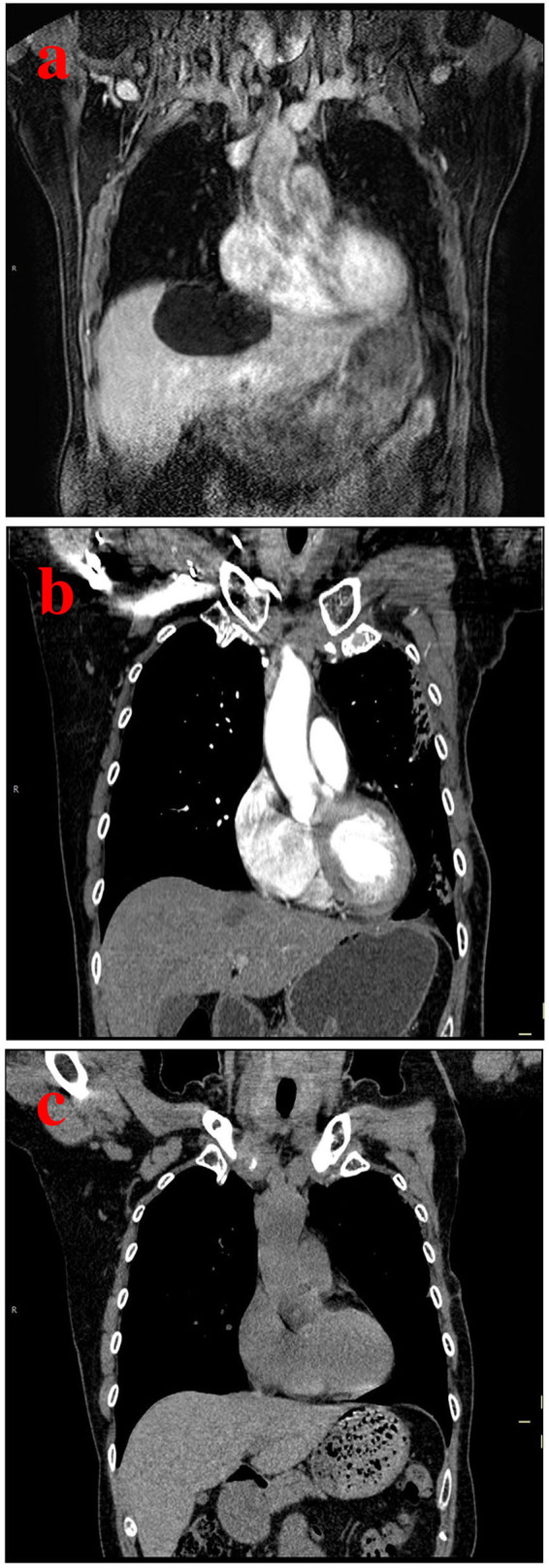
A case of a 37-year-old man with a hepatic cyst. **(a)** Pretreatment CT scan shows a hepatic cyst measuring 11.4 cm in length, 10.3 cm in width, and 8.9 cm in depth. **(b)** Follow-up CT scan after 3 months of sclerotherapy with single-session OK-432 shows a markedly decreased hepatic cyst. **(c)** Follow-up CT scan after 12 months shows complete regression of the hepatic cyst.

We identified 51 patients in group A and 47 patients in group B. Of these 98 patients, 81 were eligible for inclusion. The absence of baseline (*n* = 5), defects in follow-up imaging (*n* = 7), insufficient documentation (*n* = 3), and re-intervention (*n* = 2) were the main reasons for exclusion (Figure 2). The demographic clinical data of groups A and B are listed in Table 1. Both treatments were performed without any technical difficulties. Cytological and bacteriologic examinations were negative in both groups. No differences were found in patient characteristics, mean cyst volume, aspirated volume of cysts, and percentage of aspirated to calculated volume of cysts between the two groups.

**Table 1:** Demographic clinical data of patients.

**Characteristics**	**Group A**	**Group B**	***P*-value**
	**(*n* = 42)**	**(*n* = 39)**	
**Age, years**			
Mean age (y ± SD)	54.3 ± 11.7	54.6 ± 13.7	0.91
Range	30–77	25–85	
**Sex**			
Male	28 (66.7)	23 (59.0)	0.51
Female	14 (33.3)	16 (41.0)	
**Cyst vol**.			
Mean vol. (ml ± SD)	340.8 ± 225.1	363.2 ± 186.3	0.58
Range	63–1,880	55–1,614	
**No. Cyst**			
≤ 200 ml	10 (23.8)	12 (30.8)	0.77
>200 ml, <500 ml	15 (35.7)	13 (33.3)	
≥500 ml	17 (40.5)	14 (35.9)	

*SD, standard deviation, No, number; vol., volume*.

*Data are shown as n (%)*.

### Clinical Response

The clinical responses of groups A and B after 12 months of follow-up are summarized in Table 2. The overall success rates were 92.9% (39 of 42 patients) in group A and 79.5% (31 of 39 patients) in group B. There was no significant difference in the overall regression rate between group A and group B (P = 0.153).

**Table 2:** Clinical response of single-session OK-432 (group A) and multiple-session 99% ethanol (group B) sclerotherapy.

**Clinical response**	**≤200 ml**		***P-*value**	**>200 ml**, ** <500ml**		***P* value**	**≥500 ml**		***P-*value**
	**Group A**	**Group B**		**Group A**	**Group B**		**Group A**	**Group B**	
**No. of Cysts**									
	10 (23.8)	12 (30.8)		15 (35.7)	13 (33.3)		17 (40.5)	14 (35.9)	
**No. of successful treatments**			NA			0.457			0.049
100	4 (40.0)	5 (41.7)		4 (26.7)	4 (30.8)		2 (11.8)	1 (7.1)	
90–100	5 (50.0)	6 (50.0)		7 (46.7)	5 (38.5)		7 (41.2)	2 (14.3)	
80–90	1 (10.0)	1 (8.3)		2 (13.3)	1 (7.7)		5 (29.4)	2 (14.3)	
70–80	0 (0)	0 (0)		1 (6.7)	1 (7.7)		1 (5.9)	3 (21.4)	
Total	10 (100)	12 (100)		14 (93.3)	11 (84.6)		15 (88.2)	8 (57.1)	
**No. of failed treatments**									
<70	0 (0)	0 (0)		1 (6.7)	2 (15.4)		2 (11.7)	6 (42.9)	
**No. of relieved symptoms**			0.893			0.457			0.049
	9 (90.0)	11 (91.7)		14 (93.3)	11 (84.6)		15 (88.2)	8 (57.1)	

*No, number*.

*Data are shown as n (%)*.

### Cyst Reduction

All cysts smaller than 200 ml showed complete regression in groups A and B. Of the 200–500 ml cysts, the number of patients who experienced 100%, 100–90%, 90–80%, and 80–70% volume reductions were 4 (26.7%), 7 (46.7%), 2 (13.3%), and 1 (6.7%) in group A, and 4 (30.8%), 5 (38.5%), 1 (7.7%), and 1 (7.7%) in group B, respectively. Of the 500 ml or larger cysts, the number of patients who experienced 100%, 100–90%, 90–80%, and 80–70% volume reductions were 2 (11.8%), 7 (41.2%), 5 (29.4%), and 1 (5.9%) in group A, and 1 (7.1%), 2 (14.3%), 2 (14.3%), and 3 (21.4%) in group B, respectively.

### Symptomatic Relief

In both groups, symptomatic relief was associated with high efficacy of cyst reduction. At 12 months of follow-up, clinical symptoms were resolved in 38 of 42 (90.5%) patients in group A and 31 of 39 (79.5%) in group B, regardless of whether a complete or partial regression of the cyst was observed.

### Complications

Several treatment-related complications are presented in Table 3. For the incidences of pain at injection, the reported grade 1/2 adverse events were 2.4% in group A and 23.1% in group B, and the reported grade 3/4 adverse events were 0% in group A and 2.5% in group B. Significant differences were not found in the incidence of mild fever, allergy, and leukocytosis between groups A and B. In both groups, pain and fever could be controlled by using antipyretic analgesics and allergic reactions could be controlled by using antihistamines. Leucocytes returned to normal levels at 2 weeks post-procedure.

**Table 3:** Severity of complications related to single-session OK-432 (group A) and multiple-session 99% ethanol (group B) sclerotherapy.

**Complications^a^**	**Group A**		**Group B**	
	**Grade 1/2**	**Grade 3/4**	**Grade 1/2**	**Grade 3/4**
Pain at injection sites	1 (2.4)	0	9 (23.1)	1 (2.5)
Fever	1 (2.4)	0	2 (5.1)	0
Allergy	2 (4.8)	0	2 (5.1)	0
Leukocytosis	3 (7.1)	0	2 (5.1)	0

a * Severity of complications divided into four degrees, with grade 4 being the most severe*.

*Data are shown as n (%)*.

## Discussion

The primary finding of this study was that sclerotherapy with single-session OK-432 provided satisfactory results in the efficacy rate of cyst reduction and clinical relief without causing more adverse events when compared with multiple-session 99% ethanol.

Ethanol is the most commonly used sclerosing agent for symptomatic simple hepatic cysts (1). However, the efficacy and safety of ethanol sclerotherapy are significantly different among studies (6, 9, 12). This discrepancy may be partly explained by the number of ethanol sessions used. Several authors have suggested a lower rate of 10–68% complete resolution with a single session of ethanol sclerotherapy (18–20). Complete cyst regression improved to 73–100% with multiple sessions of ethanol sclerotherapy (9, 18). Based on these results, sclerotherapy with multiple-session 99% ethanol has been used to treat symptomatic simple hepatic cysts in our hospital. However, other authors observed an increase in the incidence of adverse events, particularly ethanol intoxication and pain, after multiple sessions of ethanol sclerotherapy (13, 18). Therefore, ethanol sclerotherapy for symptomatic simple hepatic cysts remains to be determined.

OK-432 is a lyophilized mixture of the low-virulence Su strain of *S. pyogenes* (14). Intralesional injection of OK-432 has been proven safe and effective in treating cystic osteitis fibrosa, cystic hygroma, and malignant exudate in the thoracic cavity (16). The mechanism of the effect of OK-432 is probably due to the damage to the endothelial lining, which causes obliteration of the cavity and prevents further accumulation of fluid in the lesion (15). To our knowledge, no studies about scarring of the surrounding tissue or disturbance in function after OK-432 treatment have been reported.

In our study, we compared the efficacy and safety of single-session OK-432 sclerotherapy and multiple-session 99% ethanol sclerotherapy for symptomatic simple hepatic cysts. We performed both sclerotherapies without technical difficulties but repeat injection of ethanol was a time-consuming procedure. In addition, there was a considerable risk of ethanol leakage if the pigtail catheter was displaced, although the position of the pigtail catheter was confirmed before each injection. OK-432 only damages the cyst lining and causes adhesion of the cyst wall, whereas ethanol diffuses through the cyst fluid and penetrates beyond the thin endothelial cyst lining to produce indurations of the cyst wall and unpredictable scarring. In OK-432 administration, drainage was not required after injection. In our preliminary data, the overall success rates of single-session OK-432 sclerotherapy were similar to those of multiple-session ethanol sclerotherapy. Therefore, we obtained a comparable success rate by a single injection of OK-432 instead of repeat ethanol instillation. We also found that the OK-432 group showed a higher regression rate in cysts ≥200 ml than in the ethanol group, but both treatment groups achieved complete regression in cysts <200 ml. Although the clinical response of the OK-432 group was more prominent than that of the ethanol group in cysts ≥200 ml with respect to time of exposure to the sclerosing agent, concentration, and volume of the sclerosing agent. This result implies that patients with large-volume hepatic cysts proved to be excellent candidates for single-session OK-432.

Previous studies have described ethanol as a cheap, safe, and widely available sclerosing agent, but its adverse effects, such as ethanol intoxication and pain, cannot be ignored (1, 10). The use of OK-432 as a sclerosing agent was initiated to reduce the adverse events observed after the instillation of ethanol. In our study, the pain was less frequent in the OK-432 group than in the ethanol group. Ethanol intoxication did not occur, which may be explained by the maximal volume of 50 ml of ethanol in a single injection and short sclerotherapy duration of 20 min. Although OK-432 is more expensive than 99% ethanol, the OK-432 group did not require repeat procedures and had shorter hospitalization, consequently saving on overall hospital costs. As treatment responses between OK-432 and ethanol were highly comparable, we could argue that OK-432 should be preferred as a sclerosing agent. To draw definite conclusions, prospective comparison between the two agents is required.

Our findings should be considered in the context of the limitations of this study. This was a retrospective study with small sample size. The exclusion of patients may have led to a selection bias. Large sample sizes and randomized controlled trials are required for further analysis. A direct comparison between the two groups should be performed with caution. We think the comparison in our study may be reasonable because we determined a successful treatment at 1 year of follow-up. A randomized controlled trial with larger patient groups, especially for large hepatic cysts, is warranted for further analysis.

In conclusion, our study results demonstrate that single-session OK-432 sclerotherapy is an effective and safe treatment for symptomatic simple hepatic cysts. Single-session OK-432 sclerotherapy was safer and more effective than multiple-session 99% ethanol sclerotherapy for treating large cysts, although both treatments had similar effects on small cysts.

## Data Availability Statement

The original contributions presented in the study are included in the article/supplementary material, further inquiries can be directed to the corresponding authors.

## Ethics Statement

Written informed consent was obtained from the individual(s) for the publication of any potentially identifiable images or data included in this article.

## Author Contributions

ZM and WZ conceived and designed this study. XC and WZ performed all the sclerotherapy procedures. FY, JH, and LL analyzed the data and drafted the manuscript. QM and QG were the study coordinators. All authors read and approved the final manuscript.

## Conflict of Interest

The authors declare that the research was conducted in the absence of any commercial or financial relationships that could be construed as a potential conflict of interest.

## Publisher's Note

All claims expressed in this article are solely those of the authors and do not necessarily represent those of their affiliated organizations, or those of the publisher, the editors and the reviewers. Any product that may be evaluated in this article, or claim that may be made by its manufacturer, is not guaranteed or endorsed by the publisher.

## Abbreviations

CT: computed tomography.

## References

[1] Marrero JA Ahn J Rajender Reddy K. Americal College of Gastroenterology. ACG clinical guideline: the diagnosis and management of focal liver lesions. Am J Gastroenterol. (2014) 109:1328–47; quiz 1348. 10.1038/ajg.2014.213 25135008

[2] Everson GT Emmett M Brown WR Redmond P Thickman D. Functional similarities of hepatic cystic and biliary epithelium: studies of fluid constituents and *in vivo* secretion in response to secretin. Hepatology. (1990) 11:557–65. 10.1002/hep.1840110406 1970324

[3] Macedo FI. Current management of noninfectious hepatic cystic lesions: a review of the literature. World J Hepatol. (2013) 5:462–9. 10.4254/wjh.v5.i9.462 24073297 PMC3782683

[4] Lantinga MA Gevers TJ Drenth JP. Evaluation of hepatic cystic lesions. World J Gastroenterol. (2013) 19:3543–54. 10.3748/wjg.v19.i23.3543 23801855 PMC3691048

[5] Wijnands TF Schoenemeier B Potthoff A Gevers TJ Groenewoud H Gebel MJ. Ethanol sclerotherapy or polidocanol sclerotherapy for symptomatic hepatic cysts. United European Gastroenterol J. (2018) 6:919–25. 10.1177/2050640618764940 30023070 PMC6047294

[6] Wijnands TF Görtjes AP Gevers TJ Jenniskens SF Kool LJ Potthoff A. Efficacy and safety of aspiration sclerotherapy of simple hepatic cysts: a systematic review. AJR Am J Roentgenol. (2017) 208:201–7. 10.2214/AJR.16.16130 27824501

[7] Jusufovic R Zerem E. Percutaneous treatment of symptomatic non-parasitic benign liver cysts with 20% NaCl solution. Med Arh. (2011)65:35–7. 21534451

[8] D'Agnolo HM Kievit W Takkenberg RB Riaño I Bujanda L Neijenhuis MK. Ursodeoxycholic acid in advanced polycystic liver disease: a phase 2 multicenter randomized controlled trial. J Hepatol. (2016) 65:601–7. 10.1016/j.jhep.2016.05.009 27212247

[9] Neijenhuis MK Wijnands TFM Kievit W Ronot M Gevers TJG Drenth JPH. Symptom relief and not cyst reduction determines treatment success in aspiration sclerotherapy of hepatic cysts. Eur Radiol. (2019) 29:3062–8. 10.1007/s00330-018-5851-y 30542749 PMC6510865

[10] Dietrich CF Chiorean L Potthoff A Ignee A Cui X Sparchez Z. Percutaneous sclerotherapy of liver and renal cysts, comments on the EFSUMB guidelines. Z Gastroenterol. (2016) 54:155–66. 10.1055/s-0041-106594 26854836

[11] Larssen TB Rosendahl K Horn A Jensen DK Rørvik J. Single-session alcohol sclerotherapy in symptomatic benign hepatic cysts performed with a time of exposure to alcohol of 10 min: initial results. Eur Radiol. (2003) 13:2627–32. 10.1007/s00330-003-1923-7 12955449

[12] Yu JH Du Y Li Y Yang HF Xu XX Zheng HJ. Effectiveness of CT-guided sclerotherapy with estimated ethanol concentration for treatment of symptomatic simple hepatic cysts. Clin Res Hepatol Gastroenterol. (2014) 38:190–4. 10.1016/j.clinre.2013.09.008 24210773

[13] Benzimra J Ronot M Fuks D Abdel-Rehim M Sibert A Farges O. Hepatic cysts treated with percutaneous ethanol sclerotherapy: time to extend the indications to haemorrhagic cysts and polycystic liver disease. Eur Radiol. (2014) 24:1030–8. 10.1007/s00330-014-3117-x 24563160

[14] Poldervaart MT Breugem CC Speleman L Pasmans S. Treatment of lymphatic malformations with OK-432 (picibanil): review of the literature. J Craniofac Surg. (2009) 20:1159–62. 10.1097/SCS.0b013e3181abb249 19553857

[15] Ogita K Taguchi T Suita S. Experimental study concerning safety dosage of OK-432 for intrauterine treatment. Asian J Surg. (2006) 29:202–6. 10.1016/S1015-9584(09)60088-9 16877226

[16] Ohta N Fukase S Watanabe T Ito T Aoyagi M. Effects and mechanism of OK-432 therapy in various neck cystic lesions. Acta Otolaryngol. (2010) 130:1287–92. 10.3109/00016489.2010.483480 20450399

[17] Tachibana T Kariya S Orita Y Makino T Haruna T Matsuyama Y. The efficacy of OK-432 sclerotherapy on thyroglossal duct cyst and the influence on a subsequent surgical procedure. Acta Otolaryngol. (2019) 139:788–92. 10.1080/00016489.2019.1633019 31271329

[18] Yan-Hong F Lin-Xue Q Hai-Ma G Qing Z Yu G Xiangdong H. Sclerotherapy of simple hepatic cysts by repeated aspiration and alcohol instillation. Turk J Gastroenterol. (2012) 23:359–65. 10.4318/tjg.2012.0349 22965507

[19] Zerem E Imamović G Omerović S. Percutaneous treatment of symptomatic non-parasitic benign liver cysts: single-session alcohol sclerotherapy versus prolonged catheter drainage with negative pressure. Eur Radiol. (2008) 18:400–6. 10.1007/s00330-007-0760-5 17899104

[20] Yang CF Liang HL Pan HB Lin YH Mok KT Lo GH. Single-session prolonged alcohol-retention sclerotherapy for large hepatic cysts. AJR Am J Roentgenol. (2006) 187:940–3. 10.2214/AJR.05.0621 16985138

